# Relative Age Effect in Attention Deficit/Hyperactivity Disorder at Various Stages of the Medicalization Process

**DOI:** 10.3390/children9060889

**Published:** 2022-06-15

**Authors:** Marie-Christine Brault, Emma Degroote, Mireille Jean, Mieke Van Houtte

**Affiliations:** 1 Department of Humanities and Social Sciences, VISAJ Research Chair, University of Québec in Chicoutimi, Chicoutimi, QC G7H 2B1, Canada; 2 Department of Sociology, Research Team CuDOS, Gent University, 9000 Gent, Belgium; emma.degroote@ugent.be (E.D.); mieke.vanhoutte@ugent.be (M.V.H.); 3 Department of Health Sciences, VISAJ Research Chair, University of Québec in Chicoutimi, Chicoutimi, QC G7H 2B1, Canada; mireille.jean@umontreal.ca

**Keywords:** Attention Deficit/Hyperactivity Disorder (ADHD), children, medicalization, relative age effect, teachers, parents

## Abstract

Diagnosis and pharmacological treatment of ADHD are more common among the youngest children in a classroom, born in the months immediately preceding the school entry cutoff date. The mechanisms behind this phenomenon, called the relative age effect (RAE), are not yet well understood. Nearly all hypotheses involve the school system, various teachers’ actions, and concern children’s immaturity. However, most previous studies have been based on reports of health professionals’ diagnoses and prescriptions found in official databases rather than on reports of teachers’ behavior identification or suspicion of ADHD, despite their being at the first stages of the medicalization process. Our study overcomes this limitation by using reports of parents’ and teachers’ behavior identification or suspicion of ADHD within a three-level multilevel survey design, comprising 1294 children, 130 teachers, and 17 elementary public schools. The goal of our study was to investigate whether RAE stems (1) from adults’ judgement of the child’s expression of immaturity or (2) from the consequences associated with the child’s double burden of being immature and exhibiting ADHD behaviors. Our multilevel analyses put forward the first hypothesis only, supporting the medicalization of immaturity. RAE in ADHD seems mostly initiated by teachers’ pre-diagnostic actions toward younger children.

## 1. Introduction

Attention Deficit/Hyperactivity Disorder (ADHD) is a psychiatric diagnosis characterized by abnormal levels of hyperactivity, impulsivity, and inattention expressed in at least two settings (usually home and school; [1]). With a worldwide prevalence of about 6 to 7% for children, ADHD is one of the most diagnosed conditions, with a peak for elementary school-aged children (6–12 years old, 11.4%) and preschoolers (3–5 years old, 10.5%; [2]). The predominant biomedical conceptualization of ADHD promotes the idea of an individual neurological deficit, and most ADHD treatments are pharmacological [3].

Nevertheless, the absence of a clear-cut test indicating the presence or absence of ADHD in children and the fact that ADHD symptoms are common children’s behaviors open the door to identification and labeling errors, in addition to questions concerning the validity of the diagnosis [4,5,6]. From a sociological perspective, ADHD is one of the best examples of the medicalization of deviant behaviors [4]. Medicalization is a collective definitional process, in which non-medical problems are transformed into medical problems in a series of actions where a variety of actors working both inside and outside the medical field (school, family, media, etc.) participate [4]. It raises concerns about overdiagnosis [7,8], which consists of “making people patients unnecessarily, by identifying problems that were never going to cause harm or by medicalizing ordinary life experiences through expanded definitions of diseases” [9]. In any case, these social problems must be addressed because, in addition to significant medical, pharmacological, and societal costs, children themselves experience permanent developmental consequences [7,8,10,11].

Among the reasons for the misdiagnosis and overdiagnosis assumptions is the unequal distribution of ADHD-related symptoms, diagnosis, and medication, according to children’s specific social characteristics. Many studies have shown, for example, that youth from low socioeconomic backgrounds and boys have increased probabilities of being diagnosed with ADHD and prescribed medication to manage their symptoms [2,12,13]. Inequalities have also been observed in children’s birth months relative to the school entry cutoff date. Several empirical studies, systematic reviews, and meta-analyses from around the world have shown that, compared with their oldest classmates, the youngest students—those born in the months immediately preceding the school entry cutoff date—were overrepresented among children identified as having an ADHD diagnosis and taking ADHD medication [14,15,16]. The mechanisms behind this phenomenon are not yet well understood. Among the hypotheses advanced, most studies target children’s immaturity, described as a lag in cognitive, behavioral, or emotional development. Nevertheless, they imply different and sometimes contradictory underlying mechanisms [16,17]. Because these hypotheses are associated with different implications and solutions, it seems very important to gain a better understanding of the causes of the relative age effect (RAE) in ADHD, which may in turn help explain ADHD overdiagnosis. We thus aimed to investigate this phenomenon and its underlying mechanisms in a sample of elementary school children from Québec (Canada). The originality of our work lies in an examination of the RAE at various stages of the medicalization process. Compared to other RAE studies that have focused mainly on ADHD diagnosis and medication intake from administrative databases [18,19], we have also included parents’ and teachers’ accounts of ADHD behaviors and suspicion, which refer to pre-diagnostic actions toward children and appear in the first stages of the medicalization process.

### 1.1. The RAE in ADHD

To determine when a child should enter school, his/her birthdate is compared with the school entry cutoff date. Up to a year can separate the youngest and oldest children in a classroom, which makes a world of difference at such a young age. Compared with their older classmates, younger students have been found to be more at risk of being diagnosed with and taking medication for ADHD. This phenomenon has been observed in various regions worldwide, including North and South America (e.g., [18,20,21,22]), Europe (e.g., [23,24,25]), the Middle East [26], Asia, and Australia [15,27].

Most of these studies have been based on reports of health professionals’ diagnoses and prescriptions found in official databases involving large samples and population-based data. Whereas their results provide clear scientific evidence of the presence of the phenomenon, they provide little information on how the process occurs. Researchers should focus rather on parents’ and teachers’ actions because the assignment of a psychiatric diagnosis starts long before and continues long after entering the doctor’s office. For example, earlier stages include the tracking and identification of what poses the problem, a description and an understanding of the problem by the means of medicine, and informal medical labeling by the entourage (e.g., parent, teacher, coach, etc.; [4]).

Parents’ and teachers’ accounts of behaviors have been considered in only a handful of studies on the RAE in ADHD [20,21,22,23]. Parents’ reports of ADHD behaviors are not consistently associated with relative age. Whereas two studies have shown a weak association with relative age or an absence thereof [20,23], the Canadian study by Chen [21] indicated that parents of children born within six months of the school entry cutoff date reported more hyperactivity and inattention behaviors at home compared with parents of older children. This was supported by Caye and colleagues [22], who have observed a linear relationship between birthday and ADHD-related behaviors as reported by parents. Differences in the items used to measure ADHD-related behaviors could explain the variations in the results. Teachers were found to contribute to increasing the risks of ADHD labeling for younger-in-class students [20]. This is not surprising because school is often the first environment where ADHD is identified [4,28,29], and past research has suggested that teachers play the role of the agent of medicalization [4] or disorder-spotter [29,30]. Teachers identify problematic behaviors in children, recognize some of the symptoms associated with ADHD, provide behavioral feedback and assessments, and share their suspicions with parents, other school actors, and physicians [4,31]. It is thus very important to understand if and how they contribute to the RAE in ADHD.

### 1.2. Explanations for the RAE in ADHD

At least six hypotheses have been proposed to explain the RAE in ADHD [16,17,32]. The following two are not relevant to our discussion. One suggests that ADHD is more common in children born during the same season due to exposure to similar ADHD-related pre-, peri-, or post-natal risks. This explanation, based on birth seasonality, reinforces the biological etiology of ADHD. It is however considered quite unlikely due to the great variation in the cutoff dates (and thus seasons) between countries that have observed the phenomenon [15,16,17,22]. Another hypothesis emphasizes the possible underdiagnosis of older-in-class students [16,20,23]. Whereas this phenomenon could be hypothesized, we chose not to explore it because our focus was rather on identifying the potential underlying mechanisms associated with medicalization and overdiagnosis.

The remaining four hypotheses share common elements but propose different mechanisms of actions. They all occur within the school system, involve various teachers’ action, and concern children’s immaturity. The first one suggests that school policies regarding the timing of school entry may explain the relationship between RAE and ADHD diagnosis. Some countries, such as Denmark, have more flexible school entry policies, which encourage some children to wait an extra year before entering school [32]. This preventive practice, called “redshirting”, may counteract the effect of immaturity by allowing children to develop at a slower pace. Consequently, this could explain why Pottegard and colleagues [32] have not observed the RAE in ADHD medication for Danish children. This hypothesis was also suggested as a solution to reduce the RAE in ADHD in countries where it has been observed [23,33].

A second hypothesis suggests that “entering school at a young age triggers ADHD” due to the younger-in-class students generally struggling to meet the demands of the classroom [6,34]. In this hypothesis, ADHD is conceptualized as an individual deficit, but the RAE is blamed on an inadequate match between individual characteristics of the students and that of the school environment. This stress-related hypothesis was confirmed by the study of Diefenbach et al. [34]: RAE was associated with ADHD-related behaviors at the end of the first grade but not at the beginning of the school year. However, this effect does not seem to endure over time because studies have shown that the RAE in ADHD is less common in adolescence and even disappears in adulthood [23].

A third hypothesis suggests that adults, mainly teachers, may mistake children’s immaturity for ADHD-related behaviors [16,20]. Immaturity refers to a development level below the expected threshold at a given age. Various dimensions of immaturity are associated with ADHD, including emotional, intellectual, behavioral, cognitive, neurocognitive, and brain immaturity [20,23,27]. The expression of immaturity encompasses a rather broad array of childish behaviors, including impulsivity; hyperactivity; inattention; and difficulties settling down, focusing in school, and meeting school demands [15,25]. This confusion between ADHD symptoms and immaturity could result from teachers using the group as a comparative benchmark when forming an opinion of a student. A drawback of this practice could be that the set of norms and expectations provided by the comparison group may not always be appropriate for younger students’ developmental levels [20]. This could lead the teacher to start a medicalization process, notably by identifying a problem and addressing it using medical vocabulary or by suggesting medical care [4]. This hypothesis certainly prevails in the literature on the RAE in ADHD. Among the evidence advanced to demonstrate its veracity are: (a) the stronger RAE when teachers, rather than parents, report the behaviors [20]; (b) the fact that RAE declines with age and disappears in adulthood [23]; and (c) the maturation lag in brain development indicating that children may simply need more time to develop [15]. Until now, given the nature of the data available from previous studies, it has been difficult to assess this hypothesis. One promising avenue would be to study the RAE at various stages of the ADHD medicalization process. For example, identification of deviant behaviors, such as hyperactivity, impulsivity, and inattention, and informal medical labeling of ADHD through teachers’ suspicions are known to lead to official medical labeling and treatment [4]. A study including these elements would provide information about when the RAE in ADHD appears and which adults in the child’s environment contribute to confusing immaturity with ADHD.

A fourth hypothesis proposes rather that the RAE in ADHD stems from a functional impairment in children created by a double burden. Whereas younger students in a class are not expected to show higher levels of ADHD behaviors than their older peers do, they are expected to have more difficulties dealing with the social and academic demands of the classroom [17,33]. Thus, regardless of their ADHD behaviors, younger-in-class students would be generally more at risk of struggling in school due to their immaturity [17]. Children with this double burden (immaturity and ADHD behaviors) would then have increased risks of experiencing school difficulties [33]. The greater prevalence of ADHD diagnosis and medication intake among younger-in-class students would simply be the consequence of teachers suggesting clinical help to support children with functional impairments due to this double burden.

**Hypothesis** **1** **(H1).** *School policies regarding the timing of school entry may explain the relationship between RAE and ADHD diagnosis.*

**Hypothesis** **2** **(H2).** *Entering school at a young age triggers ADHD.*

**Hypothesis** **3** **(H3).** *Adults’ confusion between ADHD symptoms and immaturity.*

**Hypothesis** **4** **(H4).** *The RAE in ADHD stems from a functional impairment in children created by a double burden (immaturity and ADHD behaviors).*

### 1.3. Study Objectives

The general goal of our study was to explore the conditions under which RAE in ADHD emerges. Two specific objectives were targeted, each concerning one hypothesis. The first objective was to test for the RAE as an expression of the medicalization of immaturity (**H3**) by looking for the RAE in ADHD at various stages of the medicalization process: (a) identification of ADHD-related behaviors, (b) informal medical labeling of ADHD through suspicion, and (c) official medical labeling and its associated treatment [4]. Additionally, we assessed which adults (parent, teacher, health professional) contribute to the occurrence of the medicalization of immaturity. Our second objective was to assess whether children having the double burden of being the youngest in the class and exhibiting ADHD-related behaviors (**H4**) are more frequently identified by teachers because of their greater struggle in school. The investigation of these two mechanisms, potentially underlying the RAE in ADHD, would provide some insights into whether RAE stems from a problem with the child’s maturity level itself (**H4**) or from adults’ judgement of the child’s expression of immaturity (**H3**). This could lead to appropriate measures to reduce overdiagnosis.

## 2. Materials and Methods

### 2.1. Data Collection

In 2017–2018 (*n* = 9) and 2018–2019 (*n* = 8), 17 elementary public schools from the province of Québec agreed to participate in a study aiming to understand the role played by schools and teachers in the identification of ADHD-related behaviors in children and their labeling as ADHD. These schools were randomly selected based on a list of stratified characteristics, such as their socioeconomic status (SES), location, and size. To recruit them, we created three lists of schools, approached the first round of schools from the first list, and when one declined, we consulted the second list, and then the third list after a second refusal. We selected schools that differed in size, SES, and location to reflect the diversity of the school environments in the region. The student populations varied from 53 to 401 enrolled students per school (M = 196.76; SD = 90.34) and the teacher population, from 6 to 34 teachers per school (M = 17; SD = 6.4). The schools’ SES varied from 3 (somewhat privileged) to 10 (underprivileged) on the Québec’s official decile rank of school SES (M = 7.71; SD = 2.37; [35]). School principals, teachers, and parents, after giving their consent to participate (approved by the University of Québec in Chicoutimi’s institutional review board), completed paper-pencil or web questionnaires, which provided information on parents’, teachers’, and health professionals’ reports of ADHD-related behaviors, suspicion, diagnosis, and medication. These variables covered several stages of the medicalization process (identification of deviant behaviors, informal labeling of ADHD through suspicion, formal labeling via the medical diagnosis, and medication; [4]). The data collection took place some months after the school starts, precisely between November and March, to allow teachers to know their students.

### 2.2. Sample

Parents/Students. Every student in the school was asked to take home a letter explaining the study, the consent form, and the short survey designed to collect the family’s SES and the child’s characteristics, such as ADHD diagnosis and medication intake. The person most knowledgeable about the child, parents in 97% of the cases, returned their questionnaire and their consent, agreeing that their child’s name be included in the teacher’s questionnaire. Data were collected for 1783 children from kindergarten to Grade 6, for a participation rate of about 60%.

Teachers. All teachers in charge of a class from kindergarten to Grade 6 were invited to complete an online questionnaire on their knowledge and beliefs about ADHD, general expectations of students, level of self-efficacy, and behavioral management practices, as well as a set of 10 questions for each student on their list. The participation rate was 77% (130 teachers). Almost all were women (*n* = 122; 94%), worked full-time (*n* = 128; 98%), had accumulated many years of experience as a teacher (M = 19.07; SD = 9.19), and had worked many years in the participating schools (M = 8.08; SD = 8.79). Between 2 and 19 teachers per school (M = 9.88; SD = 4.68) completed the survey and gave answers for 3 to 21 of their students (M = 11; SD = 3.53), for a total of 1409 students.

Study sample. The study sample consisted of the children for whom we received parents’ and teachers’ answers (*n* = 1395), minus 101 students with missing data on gender, birth month, school cycle or family’s SES. Thus, the final sample comprised 1294 students. Table 1 displays the students’ characteristics and shows descriptive information for each of the seven ADHD-related outcomes and their distribution for each birth month.

### 2.3. Variables

#### 2.3.1. ADHD-Related Outcomes

ADHD-related behaviors. Teachers reported their perceptions of the child’s inattention and agitation levels on a scale of 0 (very low) to 4 (very high). These questions were mandatory in the online questionnaire, so there were no missing data. We summed the scores for inattention and hyperactivity to form a single variable measuring ADHD-related behaviors. The scale ranged from 0 (very low) to 8 (very high); the mean was 2.91 (SD = 2.06).

Parents’ suspicion of ADHD. Both parents and teachers reported their suspicion of ADHD in the children. After indicating in a previous question that their child did not have an official ADHD diagnosis, parents were asked, “Do you suspect your child of having ADHD?”. Parents replied yes for 130 (13%) of the students.

Teachers’ suspicion of ADHD. After indicating that the child did not have an ADHD diagnosis from a doctor or health professional, teachers were asked to report whether they suspected ADHD. Teachers and parents agreed in 93% of the cases on who had an ADHD diagnosis. When the parent did not report an ADHD diagnosis for their child but the teacher did (*n* = 35), we considered that the teacher suspected ADHD. These cases were then added to the number of children suspected of having ADHD by their teachers (*n* = 181, 18%). Thus, teachers suspected 216 (21%) children of having ADHD. Parents’ and teachers’ suspicions were binary variables (0 = no; 1 = yes).

ADHD medication benefit. Teachers were asked which children, among those not already taking medication, they thought would benefit from ADHD medication. A total of 180 children (18%) were identified. Besides the 153 reported through this question, another 27 (2%) were included because, when the parent did not report their child as taking medication but the teacher did, we considered that the teacher thought ADHD medication would benefit that child.

Teacher having mentioned ADHD to parents. Parents answered the following question: “Since your child started elementary school, has a teacher ever told you that your child may have behaviors resembling ADHD symptoms?” This question concerned the teachers’ willingness to discuss their suspicions with the parents. The answer was affirmative in 22% (*n* = 280) of the cases.

ADHD diagnosis and medication. Parents were asked whether their child had received an official ADHD diagnosis from a health professional and was taking ADHD medication. We considered their responses to concern health professionals’ practices rather than their own perceptions. Therefore, we used parents’ reports of diagnosis and medication intake as a proxy for health professionals’ views and for the prevalence of ADHD diagnoses and medication intake in the sample. Table 1 shows that about a fifth of the children in our sample had received an ADHD diagnosis from a health professional (*n* = 269, 21%) and that 90% of them (*n* = 248, 19.3%) were also taking ADHD medication.

#### 2.3.2. Academic Struggling

Parents were asked if they believed their child was behind, on time, or ahead relative to the normal curriculum and if they would describe their child’s academic achievement as lower, average, or higher compared with their classmates. These two questions were combined into a variable measuring academic struggle. All children whose parents said they were below average or behind relative to the normal curriculum were considered to be struggling in school and were assigned a value of 1. Students with an average or high score received a value of 0. For four students, information for this variable was missing. Parents of 17.4% (*n* = 225) of the students indicated that their child was struggling in school.

#### 2.3.3. Relative Age Variable

Parents reported their child’s birth month. The distribution of children within birth months varied from 6.3% in February to 9.8% in August, but this difference was not statistically significant (df = 11; χ^2^ = 17.88; *p* = 0.085). The independent variable student’s relative age provided the interval between the child’s birth month and the month of October. In Québec, the law states that children must start school at the beginning of the school year in the year they turn 5 before 30 September. Thus, 1 October is the cutoff point differentiating the oldest from the youngest children. We created a continuous variable where children born in October had a score of zero, and those born in September had a score of 11: the oldest children had the lowest scores, and the youngest had the highest.

#### 2.3.4. Control Variables

Students’ socioeconomic background is a multidimensional concept [36]. To decrease the risk of overestimating its effect, it is recommended to measure SES using multiple types of indicators (e.g., economic, cognitive, and cultural capital indicators; [36]). Therefore, the students’ SES was based on three parent-reported variables: family wealth, parental occupation, and parent’s education level. Family wealth was estimated through the family’s material possessions (televisions, cars, number of bathrooms, etc.). Parents’ level of occupational prestige reflected the highest scoring occupation on the International Socio-Economic Index (ISEI), based on the International Classification of Occupations (ISCO; [37]). Parents’ education level was measured according to the highest education level attained by one of the parents, from high school to university. The students’ SES variable was standardized and reversed, so a higher score was associated with a lower family SES. Gender (boys = 1; girl = 0) was included. School cycles were used instead of the school year because elementary school in Québec is organized in cycles: we have kindergarten (cycle 0), first and second grades (cycle 1), third and fourth grades (cycles 2) and fifth and sixth grades in cycle 3.

### 2.4. Study Design and Statistical Analysis

Our data were organized in a nested structure, with students (Level 1) nested within teachers (Level 2), who were nested within schools (Level 3). This data structure violates the independent observations assumption because people in the same school are more likely to share particularities. We, therefore, favored multilevel analysis to avoid erroneous estimations and to disentangle the within-cluster effect [38].

Statistical analyses were performed with SAS software, version 9.4 Cary, NC, USA [39]. For binary outcomes, we used the PROC GLIMMIX syntax and for the continuous one, the PROC MIXED syntax. For each outcome of Hypothesis 3, we performed independent multivariate multilevel analysis and introduced the relative age variable jointly with the control variables gender, school cycle, and SES. For Hypothesis 4, we first tested for a two-way interaction between relative age and ADHD behaviors for the struggling in school outcome. Secondly, we tested for a triple interaction between relative age, ADHD behaviors, and struggling in school for the teacher’s suspicion outcome.

## 3. Results

### 3.1. Results Associated with the Medicalization of Immaturity

The results of the fixed effects of the multivariate multilevel regression models for each ADHD-related outcome of Hypothesis 3, in relationship with the student’s relative age (RAE) variable are presented in the text. The results of the covariance parameter estimates are not shown because the goal of this study was not to look for and explain between-school and between-teacher differences in the outcomes. We used a multilevel analysis simply to control for the nested data. All the detailed results of each multilevel model are available upon request.

#### 3.1.1. RAE and Identification of ADHD-Related Behaviors

The fixed coefficient of the multivariate multilevel model for ADHD-related behaviors is significantly associated with the student’s relative age variable, even when the control variables (gender, SES, and cycle) were included in the model (df = 1160; t = 5.05; *p* < 0.001). The teacher’s perceived level of hyperactivity and inattention behaviors is increasing, on average, by 0.08-point (SE 0.02) with each birth month away from October. Being born in September (and thus being amongst the youngest in the classroom) increased the perceived ADHD-related behaviors score by an average of almost 1 point (exactly 0.88) compared to students born in October.

#### 3.1.2. RAE and Informal ADHD Medicalization

Parents suspected ADHD in 13% of the children. Suspicion was lowest for children born in October (7.2%) and highest for those born in March (17.5%), followed by August (16.3%) and September (16.4%), thus slightly higher for children born in the months closest to the school entry cutoff date. However, the results of the multivariate multilevel logistic regression were not statistically significant (t = 1.57; df = 992; *p* = 0.117) and did not confirm an RAE in suspected ADHD based on parents’ answers.

Teachers suspected ADHD in 20.9% of the children, which was significantly greater than the parents’ suspicion rate (McNemar’s test statistics = 19.69, *p* < 0.001). Whereas teacher’s suspicion was lowest for children born in October (9.3%), it was 3-times higher for the youngest children in the classroom, born between June (27.5%) and September (28.6%). Results of the multivariate multilevel logistic regressions confirmed that the student’s relative age variable was statistically significant (t = 4.23; df = 1025; *p* < 0.001) in this model. A one-unit increase on the RAE variable is associated with an odds ratio [OR] of 1.11 (95% confidence interval (CI) 1.06–1.16). Specifically, being born in September is associated with an OR of 3.03 (CI 1.81–5.07) of being suspected of ADHD by teachers.

Similarly, the student’s relative age variable was statistically significant for the outcome medication intake would be more beneficial (df = 999; t = 4.43; *p* < 0.001; OR = 1.13; CI 1.07–1.19) and the outcome teacher’s having mentioned ADHD to parents (df = 1257; t = 3.47; *p* = 0.001; OR = 1.08; CI 1.03–1.12).

#### 3.1.3. RAE and Formal Medicalization

ADHD diagnosis was reported in 16.5% of children born in October, a proportion that has almost doubled for children born in the months preceding the cutoff date: August (27.3%) and September (30.9%). The student’s relative age variable was statistically significant for this outcome (df = 1284; t = 2.27; *p* = 0.023; OR = 1.05; CI 1.01–1.10). However, the RAE phenomenon was not statistically significant for taking ADHD medication (df = 1279; t = 1.92; *p* = 0.058), despite an important difference between children born in September (29.1%) and those born in October (15.8%). Thus, younger children had a greater risk of being diagnosed with ADHD but not of being prescribed medication.

### 3.2. RAE and the Double Burden 

The results of the fixed effects of the multilevel regression models for each model testing Hypothesis 4 did not support this assumption. The analysis of the main effects showed that ADHD behaviors (df = 1282; t = −7.50; *p* < 0.001; OR = 1.36; CI 1.25–1.47) were significantly associated with struggling in school but RAE was not (df = 1282; t = −1.42; *p* = 0.155). The second step involved testing for a double interaction between relative age and ADHD behaviors in the probability of struggling in school. The results of the multivariate multilevel logistic regression analyses showed a statistically significant double interaction between ADHD-related behaviors and RAE (df = 1281; t = −2.84; *p* = 0.005). Therefore, the effect of a student’s relative age on struggling in school differs with hyperactivity and inattention behaviors. The decomposition of the interaction term showed a steady decline in the strength of the association between ADHD-related behaviors and struggling in school for each month between October (estimate = 0.518; df = 1281; t = 5.99; *p* < 0.001) and September (estimate = 0.158; df = 1281; t = 2.42; *p* = 0.016). The results then show that the effect of age on struggling becomes less strong when ADHD-related behaviors are higher. The third step of the analysis consisted of testing for a three-way interaction between relative age, ADHD behaviors, and struggling in school in the probability of teachers suspecting ADHD. Results of the multivariate multilevel logistic regression analyses showed that the triple interaction was not statistically significant (df = 1017; t = 1.17; *p* = 0.243).

## 4. Discussion

Concerns about ADHD overdiagnosis in children are becoming widely discussed in general media and the scientific literature. One argument that seems to support it is the fact that, compared with their oldest peers, younger-in-class students have higher risks of being diagnosed with ADHD and being prescribed ADHD medication [14,15,16]. Despite the consistency and similarity of the results observed, it is still not clear how to interpret the phenomenon [16,17]. The goal of our study was thus to evaluate the presence of the RAE in ADHD in a sample of elementary school children from Québec and to gain a better understanding of the underlying mechanisms. Among four possible hypotheses involving the school system, teachers’ actions and children’s immaturity, we tested two specifics ones (H3 and H4). Our findings supported the Hypothesis 3 of the medicalization of immature behaviors of younger-in-class children. They did not, however, confirm the double burden hypothesis (Hypothesis 4).

Like other studies from around the world, our results confirmed the presence of inequalities in risks for ADHD diagnosis in students born in the months immediately preceding the school entry cutoff date [15,18,20,21,23,24,26]. More specifically, our findings showed that the proportion of children with an ADHD diagnosis was almost two times higher amongst children born within the two previous months of the cutoff date compared with their oldest classmates. However, our results could not confirm the RAE for medication intake. This result was surprising because, with the exception of the Danish study by Pottegard [32], the RAE for ADHD medication is usually observed [18,20,24,27]. Québec physicians might be more cautious not to medicate younger-in-class children because of their greater awareness of the possible confusion between immaturity expressions and ADHD-related behaviors, such as hyperactivity and impulsivity. This caution might exist because Canadian researchers were among the first to publish on the RAE in ADHD almost ten years ago [18], and their results may have been considered in clinical guidelines [40]. Another explanation could be that parents of these younger-in-class children might argue against medication for what they understand to be an immaturity issue. Thus, they could decide to wait and let time does its work or favor alternative medicine, such as psychotherapy, behavioral training, meditation, or yoga [41].

Nevertheless, our results indicated that health professionals participate at the later stages of the medicalization process by formally labeling younger-in-class students. However, how does medicalization occur beforehand? Looking at the pre-diagnostic steps helps to understand the mechanisms at work here.

### 4.1. Immaturity Mistaken for ADHD

Our results showed that overrepresentation of younger children occurs at each step of the ADHD medicalization process [4] and thus starts long before their visit to the physician’s office. We also found that the process begins as soon as the teachers start assessing hyperactivity, impulsivity, and inattention behaviors, thus reinforcing Elder’s [20] conclusions. Teachers were more likely to identify higher levels of ADHD-related behaviors among children born before the school entry cutoff date. In our study, inattention and hyperactivity behaviors were both associated with the RAE in a similar way. This was our main reason for creating a combined hyperactivity-inattention variable. Whereas our data did not enable us to determine whether teachers saw these behaviors as “deviant” and problematic, it seems likely because teachers were also more likely to suspect younger students and to share their suspicion with the parents of younger-in-class children. Younger-in-class students thus seem more likely to be referred to health professionals, and consequently, the pool of them really going for a consultation is larger, which brings a greater risk of leading to a diagnosis.

Not all adults surrounding a child contribute equally to the RAE in ADHD. Parents are usually not influenced by their children’s birth month or by their relative immaturity when assessing ADHD-related behaviors [20,23] or suspecting ADHD, as our results have shown. Teachers seem to play the most central role in the pre-diagnosis steps because they help with the identification of deviant problems and with their medical labeling and suspicions, and they either bring children to consult physicians or recommend a consultation [4,28]. This could be supported by the classroom context where teachers are constantly interacting with students of different maturity levels and might unconsciously compare the youngest to the oldest might. Therefore, our results confirm the teacher’s role “as a catalyst in initiating diagnosing processes” [16] (p. 6). Nevertheless, it is important to recall that not all teachers are alike and that their practices can differ considerably. First, the random part of the multilevel analyses showed great variability in the outcomes between teachers, which indicates that not all teachers act the same regarding identification or suspicion of ADHD-related behaviors and sharing their thoughts with parents. Second, interviews with teachers in a subsequent phase of the study showed that teachers do not all share the same eagerness to engage in the medicalization of their students’ behaviors [42]. All interviewed teachers had their students’ personal and academic well-being at heart, despite some being quicker than others to use medicalization to help the child perform better or gain peer acceptance. Further studies should be conducted to see if the characteristics of the teachers and of the schools explain the variability in the judgement of deviance [4]. Certain actions could also be undertaken to limit this medicalization and further overdiagnosis. Teachers have the power to push for children’s assessment for ADHD; therefore, it is essential to make them understand the phenomenon, its consequences, and that of medicalization. They need help to readjust their actions toward the youngest children in their class [5].

In sum, whereas our study design cannot totally rule out the possibility of the involvement of other mechanisms, such as children’s ADHD being triggered once they enter the school system (**H2**) [16], our results do not point to it because if ADHD-related behaviors and ADHD were triggered by school entrance at a younger age, then parents would see this as well. Our analyses thus provided strong evidence supporting the medicalization of immaturity in younger-in-class children (**H3**).

### 4.2. The Double Burden Hypothesis

We tested another hypothesis (**H4**), which suggested that teachers were more likely to identify children having the double burden of being young in class and exhibiting ADHD behaviors because of their greater struggle in school. This hypothesis emphasized the importance of functional impairment as the main reason to seek medical care [17,34]. However, it could not be confirmed by our models, which were limited by the cross-sectional nature of the study. Younger-in-class students were definitely struggling in school, possibly due to a maturation lag in their cognitive development. However, adding hyperactivity and inattention behaviors did not seem to increase their school struggle more than it did for older children. Could this suggest that an academic struggle is a problem for youngest and oldest students but not for the same reasons? For example, the former would struggle because of their immaturity, the latter due to their ADHD behaviors. This seems consistent with the hypothesis that ADHD may be underestimated in older children [16,20] and suggests that there are other factors that combine with academic struggling to prompt teachers to start a medicalization process. At least, functional impairment may involve more than just school difficulties.

### 4.3. Strengths and Limitations

In most previous studies, the investigation of the RAE in ADHD diagnosis has been based on reports from health professionals’ diagnoses and prescriptions found in official databases. The originality of our work lies in (a) the inclusion of various adults’ viewpoints on the child and (b) an examination of various stages of the medicalization process. To our knowledge, our study is the first in which the authors checked for the presence of the RAE in parents’ and teachers’ suspicions of ADHD. This may provide insight into the mechanism underlying the RAE in official medical diagnoses, given that teachers and parents are the first to suggest ADHD in children [4,28]. This implies that the RAE in ADHD starts before the official diagnosis and pharmacological treatment is given by health professionals. Our study also contributes to the literature by reinforcing the importance of social, political, and cultural aspects underlying ADHD diagnosis and its preliminary stages [4,5]. Finally, our study strengthens the need to improve diagnosis accuracy, especially in younger children where the line between normalcy and pathology is blurred more easily because of the immaturity factor.

Nevertheless, a limitation of our study is the fact that we could not compare the perception of the various adults on every ADHD-related outcome. Most outcomes were reported by either the teachers or the parents; only suspicion of ADHD was reported by both. Future studies would benefit from a comparison of answers to common questions, which would help in understanding the role of various adults in the ADHD labeling and medicalization process. The identification of teachers’ characteristics that may be more or less associated with the medicalization process of younger-in-class students could help in building more effective preventive actions targeting teachers and other school actors [5]. Additionally, it is imperative to better understand the norms according to which teachers compare students with one another. Finally, more flexible school entry policies could be important, as demonstrated in countries where redshirting practices are allowed [32].

## 5. Conclusions

The greater risks of ADHD-related outcomes among younger-in-class students are explained mostly by the medicalization of immature behaviors, which gives strength to the fears about ADHD overdiagnosis [7,10]. Researchers should definitely continue to focus on the RAE and its basic mechanisms. A general consequence of medicalization is the individualization of a social problem, and a specific consequence in the case of ADHD is that it can lead to diminishing tolerance of childish behaviors, which may bring enduring negative consequences for children, their families, and society.

## Figures and Tables

**Table 1:** Descriptive statistics of ADHD-related outcomes and children’s characteristics by birth month (*n* = 1294).

		Oct.	Nov.	Dec.	Jan.	Feb.	Mar.	Apr.	May	June	July	Aug.	Sept.
		0	1	2	3	4	5	6	7	8	9	10	11
	*n* (%)												
**Month of birth ^a^**	1294 (100)	104 (8.0)	100 (7.7)	118 (9.1)	106 (8.2)	81 (6.3)	105 (8.1)	100 (7.7)	97 (7.5)	126 (9.7)	117 (9.0)	128 (9.9)	112 (8.7)
**Boys**	649 (50.2)	51 (49.0)	51 (51.0)	60 (50.8)	56 (52.8)	39 (48.2)	47 (44.8)	45 (45.0)	53 (54.6)	65 (51.6)	63 (53.9)	59 (46.1)	60 (53.6)
**Cycle K**	216 (16.7)	19 (18.3)	18 (18.0)	23 (19.5)	15 (14.2)	9 (11.1)	15 (14.3)	19 (19.0)	23 (23.7)	20 (15.9)	19 (16.2)	18 (14.1)	18 (16.1)
**Cycle 1**	306 (23.7)	23 (22.1)	25 (25.0)	32 (27.1)	30 (28.3)	23 (28.4)	24 (22.9)	19 (19.0)	20 (20.6)	30 (23.8)	36 (30.8)	20 (15.6)	24 (21.4)
**Cycle 2**	399 (30.1)	39 (37.5)	33 (33.0)	35 (29.7)	24 (22.6)	23 (28.4)	31 (29.5)	37 (37.0)	22 (22.7)	37 (29.4)	36 (30.8)	50 (39.1)	32 (28.6)
**Cycle 3**	373 (28.8)	23 (22.1)	24 (24.0)	28 (23.7)	37 (34.9)	26 (32.1)	35 (33.3)	25 (25.0)	32 (33.0)	39 (31.0)	26 (22.2)	40 (31.2)	38 (33.9)
**Struggling in school**	225 (17.4)	12 (11.7)	14 (14.0)	18 (15.4)	20 (18.9)	9 (11.1)	16 (15.2)	23 (23.0)	12 (12.4)	22 (17.5)	20 (17.1)	30 (23.4)	29 (26.4)
**ADHD diagnosis**	269 (20.8)	17 (16.5)	18 (18.0)	19 (16.1)	18 (17.0)	18 (22.2)	21 (20.0)	27 (27.0)	13 (13.4)	24 (19.1)	25 (21.4)	35 (27.3)	34 (30.9)
**ADHD medication**	248 (19.2)	16 (15.8)	16 (16.2)	18 (15.3)	17 (16.0)	16 (19.8)	20 (19.1)	26 (26.0)	12 (12.4)	22 (17.5)	23 (19.7)	30 (23.8)	32 (29.1)
**ADHD suspicion** (**parents**)	130 (12.7) ^b^	6 (7.2)	9 (11.5)	13 (13.3)	10 (11.6)	7 (11.1)	14 (17.5)	8 (11.1)	13 (15.7)	12 (12.1)	11 (12.2)	15 (16.3)	12 (16.4)
**ADHD suspicion** (**teachers**)	216 (20.9) ^c^	8 (9.3)	14 (17.1)	16 (16.2)	15 (17.7)	10 (16.4)	20 (23.5)	15 (19.2)	15 (17.6)	28 (27.5)	25 (26.0)	28 (29.2)	22 (28.6)
**Medication benefit** (**teachers**)	180 (17.9) ^d^	6 (7.0)	10 (12.4)	13 (13.4)	9 (10.7)	10 (16.1)	15 (17.9)	13 (17.3)	13 (16.3)	27 (27.3)	21 (22.6)	27 (29.0)	16 (22.2)
**Teacher mentioned ADHD**	280 (21.6)	17 (16.8)	15 (15.5)	23 (19.5)	16 (15.4)	14 (18.0)	24 (23.1)	24 (25.0)	15 (16.1)	32 (25.6)	26 (22.8)	35 (28.0)	39 (35.8)
	M (SD)												
**Hyper-inatt behaviors**	2.91 (2.1)	2.41 (1.8)	2.78 (2.0)	2.59 (2.0)	2.56 (1.8)	2.62 (2.1)	3.04 (2.0)	2.86 (2.0)	2.63 (2.0)	3.08 (2.2)	3.26 (2.1)	3.27 (2.2)	3.53 (2.1)

^a^ The distribution of children for each month of birth is not significantly different (df = 11; χ^2^ = 17.88; *p* = 0.085). ^b^ Parents answered this question only if they did not identify the child as having an ADHD diagnosis in a previous question (denominator is *n* = 1025). ^c^ Teachers answered this question only if they did not identify the child as having an ADHD diagnosis in a previous question (denominator is *n* = 1032). ^d^ Teachers answered this question only if they did not identify the child as having an ADHD medication in a previous question (denominator is *n* = 1006).
